# Morphological Characterization and Metabolomic Analysis of the Inhibitory Effects of *Pleurotus ostreatus* Mycelium on *Triticum aestivum* L. Growth and Development

**DOI:** 10.3390/plants15081232

**Published:** 2026-04-16

**Authors:** Weiliang Qi, Jianzhao Qi, Zhilong Yao, Minglei Li

**Affiliations:** 1 School of Agriculture and Bioengineering, Longdong University, Qingyang 745000, China; 2 Center of Edible Fungi, Northwest A & F University, Yangling 712100, China

**Keywords:** spent mushroom substrate, co-culture, phytotoxicity, root infection, non-targeted metabolomics

## Abstract

With the continuous expansion of *Pleurotus ostreatus* cultivation, substantial quantities of post-harvest spent mushroom substrate (SMS) are generated. Improper disposal of this organic waste poses potential threats to soil health, including contamination and ecological imbalance. Consequently, a rigorous safety assessment is indispensable to support the sustainable and agronomically viable utilization of SMS as a soil amendment. In this study, *P. ostreatus* SMS was subjected to sterilized and non-sterilized treatments, and a controlled co-culture system integrating *P. ostreatus* mycelium with wheat was established. This system facilitated a comprehensive evaluation of residual mycelium impacts on wheat growth and development at phenotypic, cytological, and non-targeted metabolomics (LC-MS) levels. Results demonstrated that direct field application of non-sterilized SMS severely compromised wheat performance, inducing root necrosis and significantly reducing grain set. Comparative experiments confirmed that non-sterilized SMS—not its sterilized counterpart—exerted pronounced phytotoxic effects, markedly inhibiting seedling growth and triggering wilting symptoms. To elucidate the temporal dynamics of mycelial interaction, wheat seedlings were inoculated with viable *P. ostreatus* mycelium and co-cultured for seven days. Under these conditions, the mean root length of the control group (10.82 cm) was approximately threefold that of the treatment group. Histopathological analysis revealed a progressive infection pattern initiating at the root apex and extending basipetally; prolonged exposure ultimately caused complete root system collapse. Scanning electron microscopy further showed extensive mycelial colonization on infected root surfaces, accompanied by characteristic cellular damage—including severe cell wall wrinkling and widespread cell death. LC-MS profiling identified 1867 annotated compounds. Comparative analysis revealed significant dysregulation of secondary metabolism, with 495 metabolites upregulated and 419 metabolites downregulated in the treatment group. Collectively, these findings provide robust evidence that unprocessed *P. ostreatus* SMS poses tangible agronomic risks upon direct soil application. This study establishes a critical scientific foundation for developing safe, evidence-based protocols for the valorization and integrated management of SMS.

## 1. Introduction

*Pleurotus ostreatus* (Jacq.) P. Kumm.—a basidiomycete fungus belonging to the phylum Basidiomycota, class Agaricomycetes, order Agaricales, and family *Pleurotaceae*—is the most widely cultivated edible mushroom globally. It is characterized by extensive cultivation areas, abundant resource availability, advanced and standardized cultivation protocols, high biological efficiency (typically 80–120%), and favorable economic returns [1]. According to official agricultural statistics, China’s annual production of *P. ostreatus* has steadily increased from 5.5994 million tonnes in 2010 to 6.1551 million tonnes in 2022. From an industrial perspective, *P. ostreatus* plays a crucial role in advancing China’s rural revitalization strategy, promoting sustained income growth for farmers, and safeguarding large-scale agricultural development and national food security—attributes conferred by its broad environmental adaptability, short growth cycle, and rich nutritional profile.

However, the rapid expansion of this industry has concurrently generated substantial volumes of spent mushroom substrate (SMS). Given that edible fungi such as *P. ostreatus* typically degrade only 40–80% of lignocellulosic components in the substrate [2], a considerable proportion of the original substrate retains its chemical and structural integrity. As reported previously, SMS primarily comprises residual lignocellulosic materials (e.g., sawdust, corn cobs, rice or wheat straw), undegraded nutrients (notably nitrogen, phosphorus, and potassium), viable or dormant fungal mycelium, and microbial metabolites produced during substrate colonization [3]. Nevertheless, the majority of SMS is still openly stockpiled or directly incinerated. This practice not only results in wastage of valuable resources and environmental degradation but also fails to harness the substrate’s inherent potential value [4]. Consequently, this represents a critical bottleneck constraining the green and sustainable development of the *P. ostreatus* industry. Therefore, resource-oriented utilization of SMS has become an imperative research priority requiring in-depth investigation.

Studies confirm that composted SMS significantly enhances soil fertility, improves soil physicochemical properties, reduces chemical fertilizer dependency, and promotes sustainable soil resource management [5]. Additionally, SMS used as a growing medium has been shown to benefit ornamental species such as *Camptotheca acuminata* and *Salvia splendens* [2]. Furthermore, SMS applied as a basal fertilizer effectively increases soil moisture and available nutrient levels, facilitating growth in crops, including tomato [6] and kale [7]. Collectively, these findings validate the agronomic feasibility of SMS recycling into agricultural systems. However, accumulating evidence indicates that unprocessed SMS—particularly when harboring viable or metabolically active mycelial residues—poses non-negligible ecological risks to soil health, aquatic environments, and ecosystem integrity [8]. In grassland ecosystems, for instance, the aggressive proliferation of residual mycelia severely suppresses native vegetation, resulting in stunted growth, reduced tillering and plant height, leaf chlorosis, and eventual plant death under prolonged exposure, thereby forming characteristic fungal fairy rings [9]. Mechanistic studies have elucidated dual phytotoxic modes of action by pathogenic mycelia: (i) direct physical penetration into root epidermal and cortical tissues, compromising cellular structural integrity; and (ii) production and rhizosphere release of allelopathic secondary metabolites, including sesquiterpenoids, cyanogenic glycosides, and polyacetylenes, which disrupt cellular redox homeostasis, inhibit root respiration, and suppress mitotic activity [10,11]. Although prior research has documented adverse effects of fungal mycelium on plant growth, the phytotoxic potential and underlying mechanisms associated with returning discarded *P. ostreatus* substrate to agricultural soil remain poorly characterized, with limited targeted investigations available.

Against this background, the present study conducted field experiments to evaluate the effects of mycelial residues on wheat growth using sterilized and non-sterilized SMS treatments. To isolate direct fungal impacts, an in vitro co-culture system comprising *P. ostreatus* mycelium and wheat seedlings was established. Integrated analyses across root morphological traits, cytological alterations, and non-targeted metabolomic (LC-MS) profiles were performed to elucidate the specific effects of mycelial residues on wheat root development. This work provides a theoretical foundation for the scientific management and rational utilization of mushroom waste residues, with significant implications for advancing sustainable practices in the edible mushroom industry, promoting agricultural resource circularity, and safeguarding farmland ecological security.

## 2. Results

### 2.1. Impact of SMS Application on Wheat Growth and Root Health

**Figure 1:**
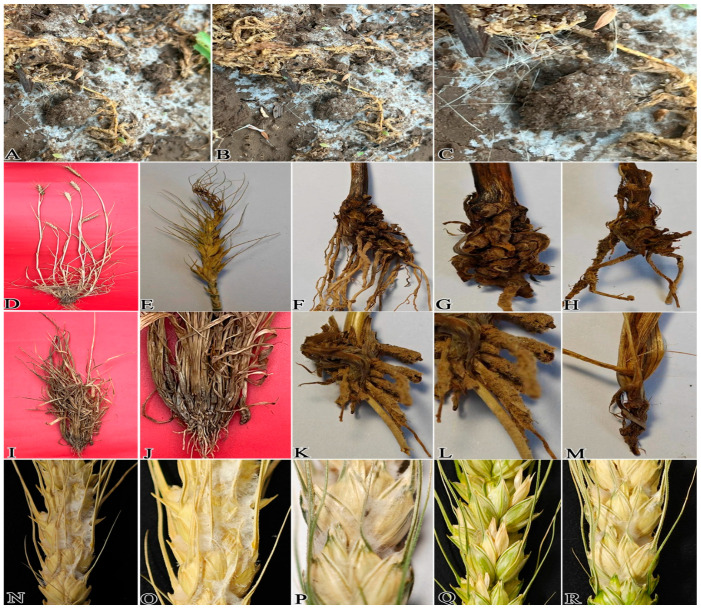
Effects of direct application of microbial rods to soil on wheat growth. (**A**–**C**) show the characteristics of soil mycelial growth after mushroom substrate incorporation; (**D**–**M**) display mycelial infection in wheat plants (**D**), ears (**E**,**N**–**R**), and roots (**F**–**M**) under mushroom substrate amendment.

**Figure 2:**
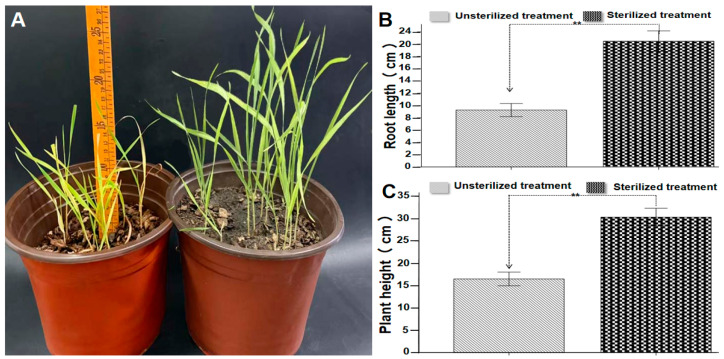
Effects of sterilized and non-sterilized treatments on *P. ostreatus* SMS (**A**), root length (**B**), and wheat plant height (**C**). Statistical analysis was performed using one-way ANOVA. Asterisks (**) indicate significant differences compared with the control: *p* < 0.01.

The results showed that while direct soil amendment with waste mushroom culture sticks improved soil looseness, a dense proliferation of white mycelia was observed on the soil surface (Figure 1A–C). During the late growth stage of wheat, the roots exhibited obvious rot symptoms (Figure 1D,F–M), accompanied by poor ear development and varying degrees of fungal infection on the ears (Figure 1E,N–R). To further verify the specific impact of the waste mushroom culture sticks on wheat growth and development, an additional experiment was conducted with two parallel treatments: soil amended with sterilized waste mushroom culture sticks and soil amended with non-sterilized waste mushroom culture sticks. The results indicated that wheat plants in the sterilized treatment grew vigorously, whereas those in the non-sterilized treatment showed significant growth inhibition. Specifically, the non-sterilized treatment group exhibited stunted growth, with leaves showing obvious chlorosis and wilting symptoms (Figure 2A). Quantitative measurements confirmed that the average plant height in the non-sterilized group was 16.51 cm (Figure 2B), which was significantly lower than that in the sterilized treatment group (*p* < 0.05). Similarly, the average root length in the non-sterilized group was 9.26 cm (Figure 2C), also significantly shorter than in the sterilized group (*p* < 0.05). These findings further confirm that non-sterilized waste mushroom culture sticks exert a significant inhibitory effect on wheat growth.

### 2.2. Verification of the Effect of P. ostreatus Mycelium on Wheat Growth and Development

**Figure 3:**
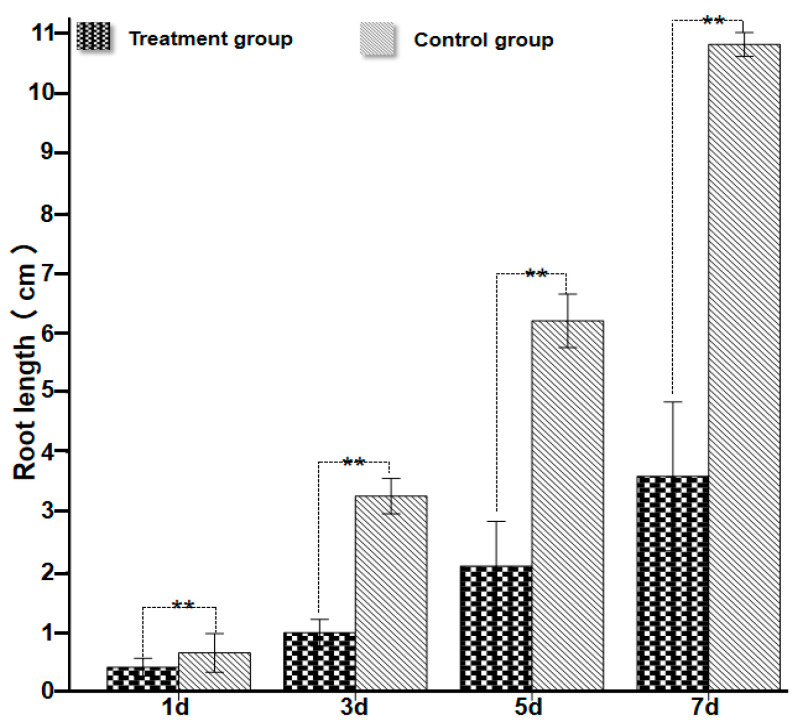
Mycelium–Wheat interaction: Effects on wheat root system growth. Statistical analysis was performed using one-way ANOVA. Asterisks (**) indicate significant differences compared with the control: *p* < 0.01.

**Figure 4:**
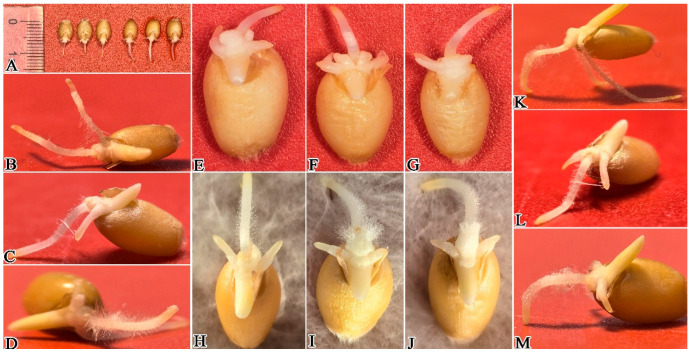
Effect of *P. ostreatus* mycelium inoculation on wheat growth and development at 1 Day post-inoculation. (**A**) shows the overall comparison between the experimental group and the control group; (**E**–**G**) represents the CK treatment; and (**B**–**D**,**H**–**M**) represent the mycelium + wheat co-culture treatment.

**Figure 5:**
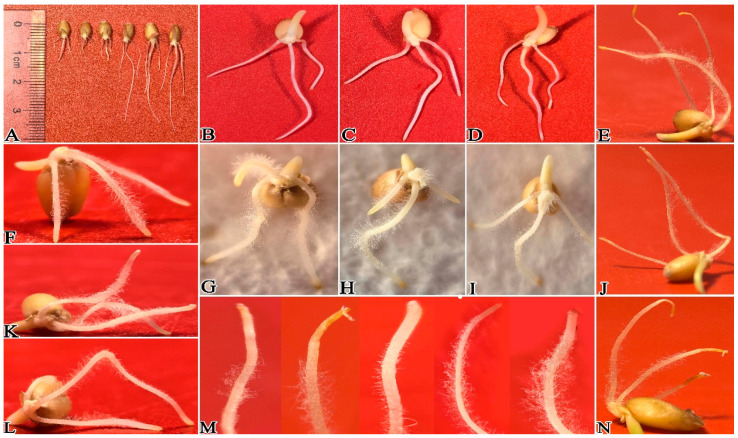
Effect of *P. ostreatus* mycelium inoculation on wheat growth and development at 3 days post-inoculation. (**A**) shows the overall comparison between the experimental group and the control group; the (**B**–**D**) group received CK treatment; and the (**E**–**N**) group underwent co-culture with mycelium and wheat.

**Figure 6:**
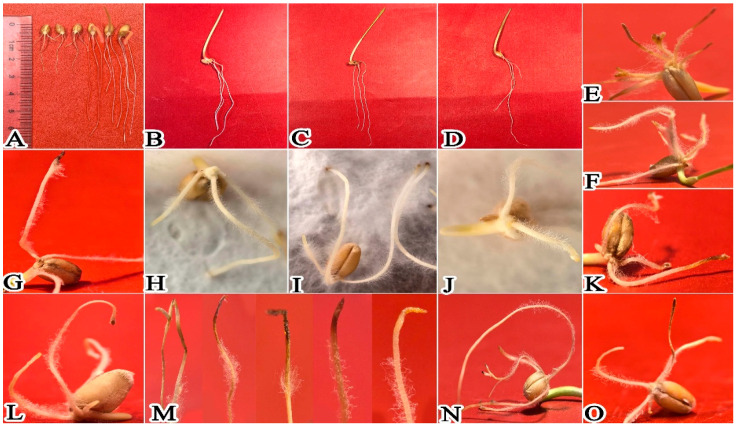
Effect of *P. ostreatus* mycelium inoculation on wheat growth and development at 5 days post-inoculation. (**A**) shows the overall comparison between the experimental group and the control group; the (**B**–**D**) group was treated with CK; and the (**E**–**O**) group was the one where mycelium was co-cultivated with wheat.

**Figure 7:**
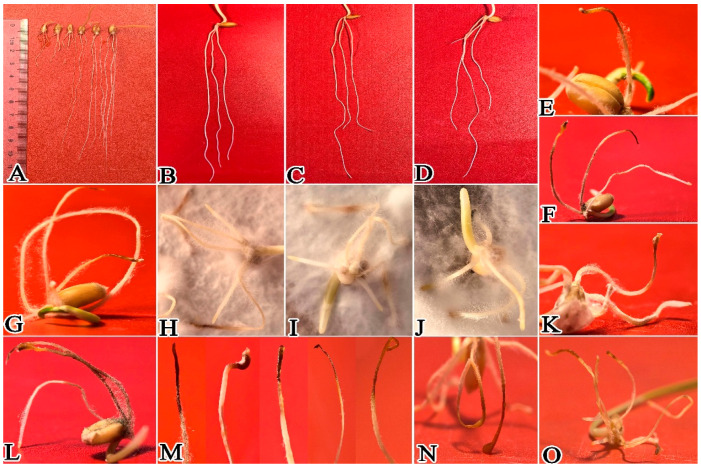
Effect of *P. ostreatus* mycelium inoculation on wheat growth and development at 7 days post-inoculation. (**A**) shows the overall comparison between the experimental group and the control group; the (**B**–**D**) group was treated with CK; and the (**E**–**O**) group was the one where mycelium was co-cultivated with wheat.

After inoculation of wheat with *P. ostreatus* mycelium culture medium, results showed that 1 day post-inoculation, *P. ostreatus* mycelium proliferated rapidly on the wheat root surface, extending from root tips toward the root bases (Figure 3 and Figure 4A–D,H–M). In contrast to the control group (Figure 4E–G), the average root length of wheat in the mycelium-inoculated treatment group was only 0.41 cm (Figure 2; Table S1), which was significantly shorter than that of the control. After 3 days of co-culture (Figure 5), the average root length of the control group reached 3.27 cm (Figure 3 and Figure 5A–D), which was 3.34 times that of the treatment group and significantly longer (*p* < 0.05). At this stage, mycelial coverage at the root tips of the treatment group decreased, but root tip cells exhibited obvious necrosis; meanwhile, a dense mycelial colonization was observed in non-necrotic root regions (Figure 5E–N). After 5 days of culture (Figure 6), wheat roots in the treatment group showed intensified wilting, whereas the control group maintained healthy growth with an average root length of 6.2 cm (Figure 3 and Figure 6A–D; Table S1)—compared to only 2.1 cm in the treatment group (Figure 6E–O). By day 7 of culture, the average root length of the control group reached 10.82 cm (Figure 3 and Figure 7A–D; Table S1), three times that of the treatment group (3.6 cm). Wheat roots infected by *P. ostreatus* mycelium exhibited extensive necrosis (Figure 7E–O). Collectively, these findings further confirm that *P. ostreatus* mycelium exerts a significant inhibitory effect on wheat root development. Additionally, the mycelium was found to follow a progressive infection pattern, expanding from root tips to root bases. Prolonged mycelial infection ultimately led to the necrosis of the entire wheat root system.

### 2.3. Impact of P. ostreatus Mycelium on Wheat Root Cell Structure

**Figure 8:**
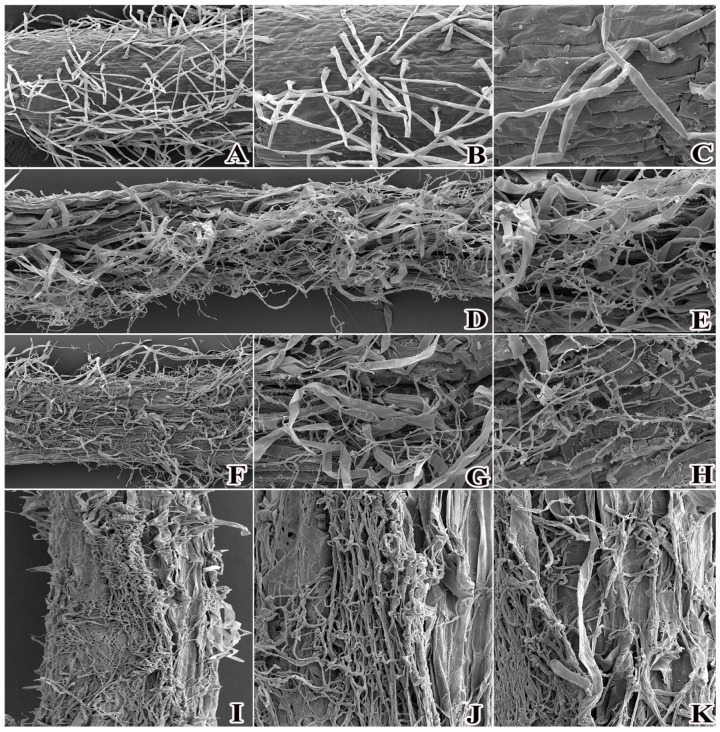
Influence of *P. ostreatus* mycelium inoculation on the cellular structure of wheat roots. (**A**–**C**) represent the CK treatment; (**D**–**K**) represent the co-culture treatment of *P. ostreatus* mycelium and wheat.

To further elucidate the effects of *P. ostreatus* mycelium on wheat root system structure, scanning electron microscopy observations were conducted on wheat roots from two groups: a control group (normal cultivation without mycelium) and a treatment group (co-cultured with *P. ostreatus* mycelium). The results showed that root surface cells of the control group were arranged regularly, with root hairs exhibiting intact morphology and a dense distribution (Figure 8A–C). In stark contrast, following co-culture with *P. ostreatus* mycelium, dense networks of *P. ostreatus* mycelia were tightly entangled around the wheat root surfaces, and root epidermal cells displayed significant shrinkage (Figure 8D–K). Additionally, wheat root hair surface cells underwent severe shrinkage: their morphology transitioned from the normal cylindrical shape to a flattened form, with obvious loss of cellular integrity. These observations further indicate that during the interaction between *P. ostreatus* mycelium and wheat roots, the mycelium inflicts substantial structural damage on wheat roots through physical entanglement and nutrient competition. This dual mechanism enables the mycelium to sequester nutrients from the roots, induces cellular water loss and shrinkage, and ultimately impairs the normal physiological functions of wheat root systems.

### 2.4. Metabolomics Data Analysis

**Figure 9:**
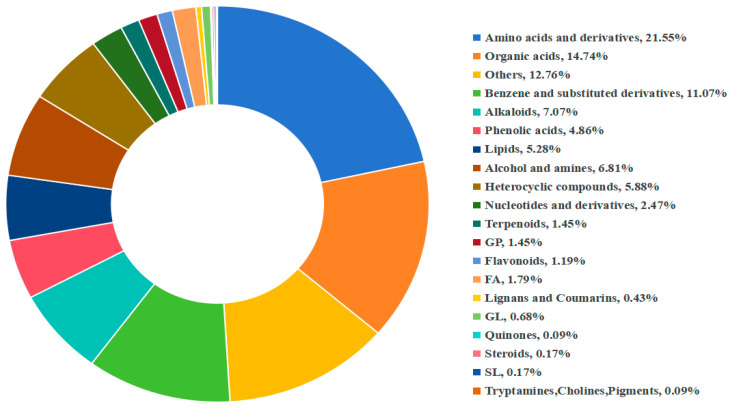
Characterization of metabolomic differentials. Ring chart of sample metabolite classifications; different colors in the figure represent distinct metabolite classifications, while the numbers indicate the proportion of metabolites belonging to each classification.

Following the co-culture, whole wheat plant samples were collected, and their LC-MS profiles were analyzed using liquid chromatography–mass spectrometry. In this study, a total of 2138 compounds were detected in the raw metabolomic data (Tables S3 and S4), which were categorized into 20 classes based on structural and functional differences (Figure 9). The major classes included: amino acids and derivatives (21.55%), Organic acids (14.74%), Others (12.76), Benzene and substituted derivatives (11.07%), Alkaloids (7.07%), Phenolic acids (4.86%), Lipids (5.28%), Alcohol and amines (6.81%), Heterocyclic compounds (5.88%), Nucleotides and derivatives (2.47%), Terpenoids (1.45%), GP (1.45%), Flavonoids (1.19%), FA (1.79%), Lignans and Coumarins (0.43%), GL (0.68%), Quinones (0.09%), Steroids (0.17%), SL (0.17%), Tryptamines, Cholines and Pigments (0.09%).

**Figure 10:**
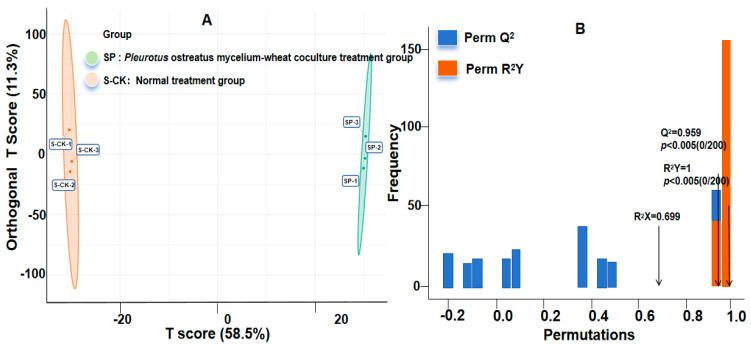
OPLS-DA score plot (**A**) and OPLS-DA validation plot (**B**).

To clarify the specific effect of *P. ostreatus* mycelium–wheat co-culture on the wheat metabolic profile, orthogonal partial least squares discriminant analysis (OPLS-DA) was performed to analyze the metabolic data of two experimental groups: the treatment group (mycelium–wheat co-culture) and the control group (wheat under normal cultivation). The results showed that the two groups of samples exhibited a clear separation trend in the OPLS-DA score plot: the three biological replicates within each group were tightly clustered, while distinct spatial separation was observed between the two groups (Figure 10A). This pattern indicates good intra-group reproducibility of the experimental samples and reliable data stability. Further validation of the OPLS-DA model yielded the following key parameters: R^2^X = 0.699, R^2^Y = 1.0, and Q^2^ = 0.959. Among these, R^2^X reflects the model’s explanatory power for the X matrix (metabolite variables), R^2^Y represents the model’s explanatory power for the Y matrix (grouping variables), and Q^2^ indicates the model’s predictive ability. These parameters demonstrate that the model possesses excellent explanatory power and predictive stability, enabling clear discrimination of metabolic differences between the treatment and control groups. Additionally, 200 permutation tests were conducted to verify the model’s reliability, further confirming its robustness and eliminating the risk of overfitting (Figure 10B).

**Figure 11:**
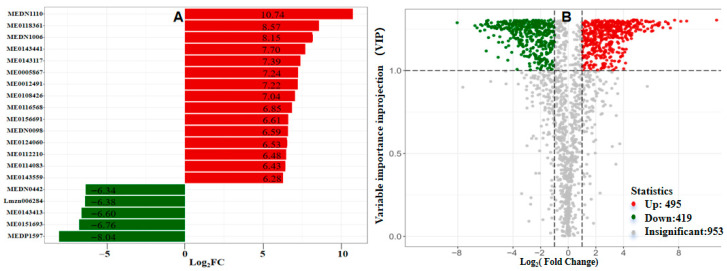
Characterization of metabolomic differentials. Fold-change bar chart (**A**) and volcano plot (**B**) of differential metabolites among different sample groups.

Differentially accumulated metabolites (DAMs) were identified between the two groups using the following criteria: Student’s *t*-test (*p* < 0.05), absolute log_2_ fold change (log_2_FC) > 1, and variable importance in projection (VIP) > 1. These DAMs were visualized via a volcano plot (Figure 11B, Tables S3 and S4). A total of 1867 compounds were detected across the treatment and control groups, among which 495 were significantly upregulated, 419 were significantly downregulated, and 953 showed no significant differences. Following qualitative and quantitative analysis of the detected metabolites, fold change differences in metabolite abundances were further compared between groups. The top 20 DAMs with the highest fold change rankings are listed below (Figure 11A; Table S2): 1,11-Undecanedicarboxylic acid (MEDN1110), 2-Carbamoylpyridine-3-carboxylic acid (ME0118361), Uric acid (MEDN1006), 4-Oxocyclohexanecarboxylic acid (ME0143441), 3-Propylmalate (ME0143117), 7-hydroxy-6-methoxy-2H-1,3-benzodioxole-5-carboxylic acid (ME0005867), 15-OxoEDE (ME0012491), N-(3-Indolylacetyl)-L-isoleucine (ME0108426), 1,2,3,6-Tetrahydrophthalimide (ME0116568), Ser-Cys-Cys (ME0156691), Picolinic acid (MEDN0098), Ethyl 2-amino-4-methylthiazole-5-carboxylate (ME0124060), Isoprenyl alcohol (ME0112210), cis-1,2-Cyclohexanediol (ME0114083), 5-amino-1H-imidazole-4-carboxylic acid (ME0143559), Pantetheine (MEDN0442), Corosolic acid (Lmzn006284), 4-Hexylphenol (ME0143413), Ile-Val (ME0151693), and Securinine (MEDP1597).

## 3. Discussion

### 3.1. Advantages and Risks of SMS Applied as Soil Amendments

The global scale of mushroom cultivation has been continuously expanding, with the global market volume projected to reach 208.4 million tons by 2026 [12,13]. As the world’s largest mushroom producer, China accounts for over 70% of the global total output. However, mushroom harvesting generates substantial quantities of SMS [14]. Previous studies have demonstrated that edible fungus-derived waste mushroom stalks are rich in organic matter and mineral nutrients, and can serve as a soil amendment to improve soil aeration, enhance water-holding capacity, and boost nutrient retention—endowing them with considerable application potential [15]. Nevertheless, SMS also contains abundant residual fungal mycelia and other pathogenic microorganisms. Improper disposal or direct application without adequate treatment may lead to soil contamination [16,17]. Therefore, conducting a comprehensive safety assessment of SMS is crucial for its safe utilization as a soil amendment [18]. Supporting the agronomic benefits, Hiroko et al. [19] reported that adding SMS from *P. ostreatus* and button mushrooms to soil significantly increases soil organic matter content, improves soil porosity, and enhances micro-aggregate formation—effectively optimizing soil physicochemical properties and exerting a positive impact on soil quality. In contrast, adverse effects have also been documented. Cheng et al. [20] found that direct soil application of SMS from shiitake mushrooms and *P. ostreatus* can induce phytotoxicity in plant roots and disrupt ecosystem balance [21]. Similarly, Kwiatkowska and Joniec [22] observed significant growth inhibition in plants following direct application of discarded SMS. To further verify these dual effects, the present study crushed SMS for direct soil application prior to wheat planting. The results showed that while soil loosening was significantly improved after stalk application, extensive white mycelial proliferation was observed in the soil over time. Correspondingly, wheat plants exhibited symptoms such as stunting, necrosis, and poor ear development. Notably, mycelial colonization was also observed on wheat ears. Collectively, these findings confirm that waste mushroom stalk application can enhance soil loosening, but concurrently poses potential risks to wheat growth. This duality is likely associated with the presence of large quantities of residual mushroom mycelia in the stalks, as well as the destructive effects of accompanying pathogenic fungi and bacteria.

### 3.2. Phytotoxic Effects of P. ostreatus Mycelium on Wheat Growth and Development

Fungi have an evolutionary history predating that of plants, and their interactions with plants are broadly categorized into two types: beneficial symbiosis and detrimental parasitism. Beneficial fungi can significantly promote plant growth and development through nutrient supply and stress tolerance regulation. In contrast, phytopathogenic fungi act as “nutrient thieves,” sequestering critical resources such as carbohydrates and minerals from host plants, disrupting normal physiological metabolism, and ultimately leading to growth retardation or even plant death [23]. Historically, Brian et al. [24] demonstrated that root growth in oats (*Avena sativa*) was significantly inhibited when grown in soil infested with *Aspergillus clavatus*. Subsequent studies confirmed that after infecting cotton roots, fungal mycelia can encircle and colonize the root system, destroying root cells and leading to necrosis [25]. Such fungal phytotoxicity is prevalent in natural ecosystems. A classic example is the “fairy ring” phenomenon, where aggressive mycelial invasion of plant roots—despite sometimes involving a form of symbiosis—severely impairs normal plant growth. This is typically characterized by stunted growth, sparse stand density, and, in severe cases, large-scale plant mortality, which eventually results in bare, unvegetated patches. These effects can cause substantial damage to the vegetation structure and stability of local ecosystems [26]. Studies have shown that returning mushroom substrate to agricultural fields can lead to the death of wheat plants, accompanied by the growth of white fungal hyphae on wheat ears. This observation indicates that mushroom hyphae exert a considerable adverse effect on wheat growth following field incorporation. To further verify the impact of mushroom hyphae on wheat growth and development, this study employed the tissue isolation method to obtain pure cultures of mushroom hyphae on PDA medium, which were subsequently inoculated onto wheat plants. After 7 days of cultivation, the results revealed a distinct infection pattern: mushroom mycelia initiated growth at the wheat root apices and gradually spread basipetally toward the root bases. In the later stages of co-culture, the wheat roots exhibited extensive necrosis. Scanning electron microscopy observations further corroborated these findings, revealing that mycelia-infected root cells displayed severe morphological damage, including dense mycelial entanglement, cell wall shrinkage, and cellular necrosis. Collectively, these results confirm that *P. ostreatus* mycelium exerts significant phytotoxic effects on wheat roots.

### 3.3. Key Metabolites Involved in the Interaction Between P. ostreatus Mycelium and Wheat

To defend against pathogenic fungal invasion, plants actively synthesize a suite of secondary metabolites [27,28], including saponins, phenolics, terpenoids, and alkaloids. These compounds can either directly inhibit fungal growth or trigger and regulate the plant immune response [29]. In a study focusing on fairy ring symptoms, Song & Tolgor [30] observed that dense fungal mycelial proliferation in soil establishes strong competitive interactions with the root systems of neighboring plants. During such bilateral interactions between fungi and host plants, both organic spent mushroom substrates synthesize and release diverse secondary metabolites with distinct structures and functions, including lipids, phenols, terpenoids, and nitrogen-containing compounds. Using a system involving fairy rings formed by *Floccularia luteovirens* [31] and dwarf juniper roots, Cao [32,33] identified 32 significantly altered secondary metabolites, of which 20 were upregulated, and 12 were downregulated. In the present study, untargeted metabolomic analysis revealed that following 7 days of co-culture between *P. ostreatus* mycelium and wheat seedlings, 495 metabolites were significantly upregulated and 419 were significantly downregulated. These results clearly demonstrate that *P. ostreatus* mycelium strongly disturbs the metabolic homeostasis of wheat and profoundly affects its growth and development.

Emerging research highlights that lipids not only form the structural framework of plant biomembranes but also act as dual-functional regulators, serving as both “signal transducers” and “defense modulators” during stress responses. For instance, lipid-mediated signal transduction pathways are rapidly activated in barley upon exposure to salt stress [34]. In the present study, the 7-day co-culture experiment between *P. ostreatus* mycelium and wheat revealed that lipids, particularly tridecanedioic acid, represented the most significantly upregulated class of secondary metabolites. This result strongly suggests that lipids exhibit a pronounced metabolic response to *P. ostreatus* exposure and are likely implicated in mediating signal transduction or physiological regulation during this interaction, thereby providing critical clues for elucidating the underlying stress resistance mechanic SMS and metabolic crosstalk in the co-culture system. Consistent with prior reports, plants are known to alter the composition of root exudates—including amino acids, organic acids, and diverse secondary metabolites—when challenged by pathogenic microorganisms in SMS. Metabolomic studies have documented significant perturbations in organic acids, amino acids, and their derivatives in *Arabidopsis thaliana* under hypoxia [35] and in cotton seedlings under salt stress [36]. Similarly, extensive organic acid production has been observed in the late stages of pea–fungus interaction [37]. Our results align with these findings, confirming that wheat infected by *P. ostreatus* mycelium exhibits significant upregulation of organic acids, amino acids, and their derivatives. Furthermore, interactions between different fungal species and plants have been shown to induce the production of various secondary metabolites, such as alkaloids, alcohols, and terpenoids [38]. The current study corroborates this pattern, demonstrating that these metabolite classes were also differentially expressed to varying degrees in wheat co-cultured with *P. ostreatus* mycelium. In summary, the co-culture system of *P. ostreatus* mycelium and wheat triggers substantial differential expression of secondary metabolites. Collectively, these findings provide novel experimental evidence for understanding the metabolic regulatory mechanic SMS governing the interaction between fungi and gramineous plants.

## 4. Materials and Methods

### 4.1. Experimental Materials

The winter wheat variety “Longyu 10” was provided by the Wheat Research Institute of Longdong University and was kept in this laboratory, with the copy number LY10.

### 4.2. Experimental Design

#### 4.2.1. Experiment of Shredded SMS Amendment and Pot Validation

(1) Field experiment with shredded SMS: The experiment was conducted in the experimental greenhouse of Longdong University. To alleviate soil compaction and poor aeration caused by long-term continuous cropping and to improve soil structure and fertility, shredded SMS was first homogenized and evenly spread over the soil surface at a rate of 500 g/m^2^. The soil was then plowed to a depth of 20–30 cm using a rotary tiller to ensure thorough mixing of the substrate with the soil. Winter wheat cv. LY 10 was sown after land preparation.
(2) Pot validation experiment: To further verify the effects of shredded SMS on wheat growth and development, two treatments were established: sterilized (autoclaved at 121 °C for 30 min) and non-sterilized. Shredded substrate from each treatment was mixed into soil (soil was sterilized at 120 °C for 30 min) in plastic pots, and wheat cv. Longyu 10 was sown. Each treatment included 12 biological replicates. Plant height and root length were measured during the experimental period to evaluate the effects of different substrate treatments on wheat growth.

#### 4.2.2. Co-Culture Experiment of *P. ostreatus* Mycelium and Wheat

To further characterize the direct effects of mushroom mycelium on wheat growth and development, the following procedures were performed:

(1) Preparation of PDA medium: Potato dextrose agar (PDA) medium was prepared using 200 g potatoes, 20 g agar, 20 g glucose, and 1000 mL distilled water. The medium was dispensed into culture bottles (20 mL per bottle), autoclaved at 121 °C for 30 min, and stored at 4 °C until use.
(2) Isolation and purification of mushroom mycelium: Fresh fruiting bodies of *P. ostreatus* were surface-disinfected with 75% ethanol. Inner mycelial tissues (approximately 0.5 cm × 0.5 cm) were excised under aseptic conditions and inoculated onto PDA medium. Inoculated plates were incubated in the dark at 25 °C until mycelium fully covered the medium surface (approximately 20 days), yielding pure mushroom mycelium for subsequent experiments.
(3) Wheat seed preparation: Healthy, mature seeds of wheat cv. Longyu 10 were washed, air-dried, vernalized at 4 °C for 12 h, and then pre-germinated for 3 days before use.
(4) Mycelium-wheat co-culture: Under aseptic conditions, 3-day-old pre-germinated wheat seedlings were transferred into culture bottles, with the treatment group comprising wheat seedlings cultured with mushroom mycelium and the control group comprising wheat seedlings cultured without mycelium. Seedlings were incubated for 7 days under controlled conditions: temperature (23 ± 1) °C, light intensity 3000 lx, and photoperiod 16 h/d.

### 4.3. Measurement Parameters

After inoculation of newly germinated wheat seedlings, root morphology was recorded at 1, 3, 5, and 7 days (one measurement every 2 days). Root length was measured and photographed. All measurements were conducted continuously for 7 days, with 60 culture bottles per replicate at each time point.

### 4.4. Scanning Electron Microscopy Observation

Samples for scanning electron microscopy (SEM) were prepared according to standard protocols. To further examine the effects of mushroom mycelium on wheat root structure, root samples from the control and treatment groups were obtained. Then, fresh wheat root tissues were cut into small pieces (approximately 5 mm × 5 mm) and immediately fixed in 2.5% glutaraldehyde at 4 °C for 12–24 h. After fixation, samples were rinsed three times with 0.1 mol/L phosphate-buffered saline (PBS, pH 7.0) for 15 min each, then dehydrated in a graded ethanol series (30%, 50%, 70%, 80%, 90%, and 100%) for 15–20 min per step, with two changes of 100% ethanol. Following dehydration, samples were dried using a critical point dryer, mounted on aluminum stubs with conductive adhesive tabs, and sputter-coated with gold for 10–20 nm using an ion sputtering device. Microscopic observations were performed using a scanning electron microscope at an accelerating voltage of 5–15 kV, and representative images were captured for analysis.

### 4.5. Non-Targeted Metabolomics

#### 4.5.1. Sample Preparation

Non-targeted metabolomic analysis was performed using liquid chromatography–mass spectrometry (LC-MS) to identify metabolites and obtain relative quantitative information in samples from the treatment (mushroom mycelium–wheat co-culture) and control groups. Statistical analyses were conducted to identify significant differences between groups, enabling interpretation of relationships between metabolites and corresponding biological processes. Whole wheat plant samples (approximately 30 g fresh weight) from the treatment and control groups were collected and homogenized in 1500 μL of pre-cooled 70% methanol–water internal standard extraction solution at −20 °C. The mixture was vortexed for 30 s every 30 min, repeated six times. After centrifugation at 12,000 rpm for 3 min, the supernatant was collected, filtered through a 0.22 μm microporous membrane, and transferred to an autosampler vial for LC-MS analysis. Each treatment included three biological replicates, yielding a total of six samples (three per group).

LC-MS analysis was performed using a Thermo Q Exactive HF-X liquid chromatography–mass spectrometry system (Thermo Fisher Scientific, San Jose, CA, USA). Chromatographic separation was achieved on a Waters ACQUITY UPLC HSS T3 column (100 mm × 2.1 mm, 1.8 μm; Waters Corporation, Milford, MA, USA) maintained at 40 °C. The mobile phase consisted of A: 0.1% formic acid in water, and B: 0.1% formic acid in acetonitrile. The flow rate was 0.4 mL/min. Mass spectrometry was operated in positive and negative electrospray ionization (ESI) modes.

#### 4.5.2. Data Processing

Raw mass spectrometry data were converted to mzML format using ProteoWizard (version 3.0) and processed using the XCMS (version 3.99) package for peak detection, alignment, and retention time correction. Peaks with a missing value rate > 50% were removed. Remaining missing values were imputed using the KNN algorithm for values < 50% and 1/5 of the minimum value for values > 50%. Peak area correction was performed using the support vector regression (SVR) method. Metabolite identification was carried out using an in-house database, integrated public libraries, predictive libraries, and the MetDNA method. Compounds with a comprehensive identification score ≥ 0.5 and a coefficient of variation (CV) < 0.5 in quality control (QC) samples were retained. Data from positive and negative ionization modes were merged by selecting compounds with the highest confidence level and lowest CV, generating the final dataset (all_sample_data.xlsx). Orthogonal partial least squares discriminant analysis (OPLS-DA) was performed using the OPLSR.Anal function in the MetaboAnalystR package within R software (version 4.5.3).

#### 4.5.3. Screening of Differential Metabolites

Differential metabolites were initially screened based on variable importance in projection (VIP) values derived from the OPLS-DA model. Univariate statistical analyses, including fold change (FC) and significance testing, were further applied. The screening criteria for differential metabolites were as follows: VIP > 1 and FC ≥ 2 or FC ≤ 0.5. Metabolites meeting these thresholds were considered significantly different between the treatment and control groups.

### 4.6. Data Analysis and Visualization

Statistical analysis was performed using SPSS 19.0. One-way analysis of variance (ANOVA) was used to assess significant differences between treatments, with *p* < 0.05 considered statistically significant. All figures were processed and prepared using Adobe Photoshop CC 2018.

## 5. Conclusions

Through multi-dimensional analyses—including phenotypic characterization, cytological observations, and metabolomic profiling—this study systematically investigated the effects of *P. ostreatus* mycelium on wheat growth and development. The key findings are as follows: Direct soil application of SMS not only promoted the extensive proliferation of white mycelia on the soil surface but also caused severe root rot and insufficient grain filling in wheat during the late growth stage. Further comparative experiments using sterilized and non-sterilized SMS confirmed that the application of non-sterilized substrate led to wheat wilting and even plant death. In the *P. ostreatus* mycelium–wheat co-culture system, *P. ostreatus* mycelia rapidly colonized the root surface upon infection and spread gradually from the root tip to the root base. With the continuous inward invasion of mycelia, wheat roots eventually exhibited extensive necrosis. Metabolomic analysis detected a total of 1867 compounds. Compared with the control group, 495 secondary metabolites were significantly upregulated and 419 were significantly downregulated in the treatment group. In conclusion, direct application of SMS to soil may pose potential risks to crop growth. Therefore, standardized sterilization treatment is essential prior to agricultural reuse of SMS, so as to ensure farmland ecological security and normal crop growth. This study provides a scientific basis and practical guidance for the rational implementation of SMS soil amendment technology.

## Data Availability

The original contributions presented in this study are included in the article/Supplementary Materials. Further inquiries can be directed to the corresponding author.

## Supplementary Materials

The following supporting information can be downloaded at: https://www.mdpi.com/article/10.3390/plants15081232/s1.
