# Supplementary material for: Morphological Characterization and Metabolomic Analysis of the Inhibitory Effects of Pleurotus ostreatus Mycelium on Triticum aestivum L. Growth and Development
Source: Plants (Basel). 2026 Apr 16;15(8):1232. doi: 10.3390/plants15081232 (PMC13119485; doi:10.3390/plants15081232)
Supplement: Supplementary file 1 [file plants-15-01232-s001.zip › plants-4218979-supplementary/Supplementary Files/supporting INFO.pdf]

Combined morphological and Metabolomic analysis to explore the effects of *Pleurotus ostreatus* mycelium on *Triticum aestivum* L. growth and development

Weiliang Qi[1*], Jianzhao Qi[2], Zhilong Yao[1], Minglei Li[2]

1 School of Agriculture and Bioengineering, Longdong University, Qingyang 745000, China;qwl9196@163.com(Q.W.); 2997617408@qq.com(Y.z.)

2 Center of Edible Fungi, Northwest A & F University, Yangling 712100, China; e-mail@e-mail.com

* Correspondence: qwl9196@163.com (Q.W.)

Table S1. Effects of Sterilized and Non-Sterilized Substrate on Wheat Growth

Experimental treatment duration	Non-sterilization treatment	Sterilization treatment
1d	0.41 ± 0.06	0.65 ± 0.13
3d	0.98 ± 0.09	3.27 ± 0.11
5d	2.10 ± 0.30	6.20 ± 0.20
7d	3.60 ± 0.50	10.82 ± 0.08

Table S2. The top 20 metabolites ranked by difference ratio

Index	Compounds	Class I	Class II	Formula	Fold_Change	Log2FC
MEDN1110	1,11-Undecanedicarboxylic acid	Lipids	Free fatty acids	C13H24O4	1712.514	10.741
ME0118361	2-Carbamoylpyridine-3-carboxylic acid	Organic acids	Organic acids	C7H6N2O3	380.914	8.573
MEDN1006	Uric acid	Nucleotides and derivatives	Nucleotides and derivatives	C5H4N4O3	284.128	8.150
ME0143441	4-Oxocyclohexanecarboxylic acid	Others	Lactones	C7H10O3	207.933	7.699
ME0143117	3-Propylmalate	Organic acids	Organic acids	C7H12O5	168.137	7.393
ME0005867	7-hydroxy-6-methoxy-2H-1,3-benzodioxole-5-carboxylic acid	Benzene and substituted derivatives	Benzene and substituted derivatives	C9H8O6	151.364	7.241
ME0012491	15-OxoEDE	Lipids	Free fatty acids	C20H34O3	149.135	7.220480
ME0108426	N-(3-Indolylacetyl)-L-isoleucine	Amino acids and derivatives	Amino acids and derivatives	C16H20N2O3	131.713	7.041
ME0116568	1,2,3,6-Tetrahydrophthalimide	Alcohol and amines	Amines	C8H9NO2	115.456	6.851
ME0156691	Ser-Cys-Cys	Amino acids and derivatives	Amino acids and derivatives	C9H17N3O5S2	97.557	6.608
MEDN0098	Picolinic acid	Organic acids	Organic acids	C6H5NO2	96.51816199	6.592728537
ME0124060	Ethyl 2-amino-4-methylthiazole-5-carboxylate	Others	Lactones	C7H10N2O2S	92.215	6.526
ME0112210	Isoprenyl alcohol	Alcohol and amines	Alcohols	C5H10O	89.166	6.478
ME0114083	cis-1,2-Cyclohexanediol	Alcohol and amines	Alcohols	C6H12O2	86.1136	6.428
ME0143559	5-amino-1H-imidazole-4-carboxylic acid	Organic acids	Organic acids	C4H5N3O2	77.689	6.279
MEDN0442	Pantetheine	Alkaloids	Alkaloids	C11H22N2O4S	0.012	-6.339
Lmzn006284	Corosolic acid	Terpenoids	Triterpene	C30H48O4	0.012	-6.377
ME0143413	4-Hexylphenol	Phenolic acids	Phenolic acids	C12H18O	0.010	-6.602
ME0151693	Ile-Val	Amino acids and derivatives	Amino acids and derivatives	C11H22N2O3	0.009	-6.757
MEDP1597	Securinine	Alkaloids	Terpenoid alkaloids	C13H15NO2	0.003	-8.044
